# Pancreatic Cystic Neoplasms: Diagnosis and Management

**DOI:** 10.3390/diagnostics13020207

**Published:** 2023-01-05

**Authors:** Abigail Schubach, Shivangi Kothari, Truptesh Kothari

**Affiliations:** 1 Department of Internal Medicine, University of Rochester Medical Center, Rochester, NY 14642, USA; 2 Department of Gastroenterology, University of Rochester Medical Center, Rochester, NY 14642, USA

**Keywords:** pancreatic cystic neoplasm, intraductal papillary mucinous neoplasm, mucinous neoplasm, next generation sequencing, endoscopic ultrasound-guided through-the-needle microforceps biopsy

## Abstract

Pancreatic cancer is one of the most lethal cancers, largely related to the difficulties with early detection, as it typically presents in later stages. Pancreatic cystic neoplasms (PCN) are commonly diagnosed as incidental findings on routine imaging. PCN is becoming more frequently detected with the increasing ease and frequency of obtaining cross-sectional images. Certain subtypes of pancreatic cysts have the potential to progress to malignancy, and therefore, clinicians are tasked with creating a patient-centered management plan. The decision of whether to undergo surgical resection or interval surveillance can be challenging given the criteria, including PCN size, pancreatic duct dilation, presence of a mural nodule, and clinical symptoms that play a potential role in risk stratification. Furthermore, the guidelines available from the major gastrointestinal societies all differ in their management recommendations. In this review, we detail an overview of the different types of PCNs and compare major guidelines for both diagnosis and management. We include emerging evidence for next-generation sequencing as well as confocal needle endomicroscopy to aid in the diagnosis and determination of malignancy potential and diagnosis.

## 1. Introduction

Pancreatic cystic neoplasms (PCN) are a category of pancreatic cysts that include both cysts of malignant and non-malignant potential. Given the different implications of the types of cysts, the correct diagnosis and optimal surveillance are essential. With the increased frequency, better quality and accessibility of cross-sectional imaging, the detection of PCN has increased, with most PCNs incidentally discovered on non-pancreatic imaging [1,2]. The incidence of pancreatic cysts in the US population is estimated to be between 3% and 15% and increases with age [3]. Along with the increased detection, there have been several different guidelines released for the surveillance and management of PCNs. These guidelines attempt to balance preventing/early detection of malignancy with avoiding unnecessary surgery, costs, and potential harm to our patients. This balance of surveillance and management of pancreas cysts remains an intimidating and daunting task for physicians. The guideline recommendations have varying amounts of evidence and have important differences, potentiating the need to summarize and review the different types and management recommendations of the most common PCNs based on current literature.

PCNs can be broadly classified as either mucinous or non-mucinous. Mucinous PCNs are lined by columnar epithelium and produce mucous. Of the mucinous PCNs, there are intraductal papillary mucinous neoplasms (IPMN) and mucinous cystic neoplasms (MCN), and of the non-mucinous neoplasms, there are serous cystic neoplasms (SCN), cystic neuroendocrine tumors (cNET), and solid pseudopapillary neoplasms (SPN). Understanding the different types of PCNs helps guide management, given mucinous cysts have a higher risk of malignant potential when compared to non-mucinous cysts.

The degree to which mucinous PCNs have malignant potential is unclear, and the available literature is somewhat contradictory, though we know that certain cysts have higher malignant potential than others. The presence of malignancy in MCN has been estimated to be 0–34%. In a study of 344 patients with MCN, 27% of cysts were malignant, but only one of the 40 malignant MCNs was less than 3 cm, confirming that with increasing size, malignancy risk also increases [4]. IPMNs have a reported 6 to 46% overall risk of transforming into pancreatic cancer, though those coming off of the main pancreatic duct have a higher risk than the branch duct, reported as 60 to 92 [5,6,7]. More recent data suggest it can be as high as 25% for high-risk IPMNs; however, natural history studies that rely on surgical specimens include a higher number of high-risk lesions than in the general population, likely overestimating the true cancer risk [8]. Furthermore, PCNs increase with age, as does comorbidity and surgical risk, therefore, the decision to enroll in a surveillance program is nuanced, and decisions must come from a patient-centered approach, considering patient preferences, surgical candidacy, and long-term health goals. 

## 2. Classification/Definition

PCNs are typically classified as either mucinous or non-mucinous, as mucinous cysts have a higher risk of pancreatic malignancy.

### 2.1. Mucinous Cystic Neoplasms

**Figure 1:**
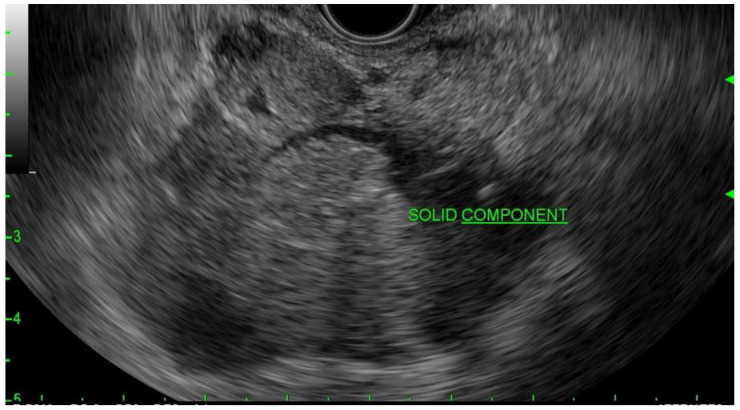
EUS image of the mural nodule (labeled solid component) in a mucinous cyst.

MCNs usually present in the fifth to seventh decade of life and are almost exclusively in females. Typical imaging characteristics consist of unilocular or septate cysts with wall calcifications (Figure 1). MCNs are defined as having ovarian-type stroma with mucin production. A solid component may suggest malignancy [9]. The aspirate is usually viscous, and the cytology findings consist of columnar cells with variable atypia. On cytology, if cells are present, there are columnar cells with stains positive for mucin. CEA is usually >200 mg/mL in approximately 75% of lesions, and the typical glucose level is usually less than fifty [10]. In a study of 90 resected MCNs, only 10% contained either high-grade dysplasia or pancreatic cancer; however, the reported rate of malignancy ranges from 0 to 34% [11,12,13,14]. 

### 2.2. Intraductal Papillary Mucinous Neoplasms

**Figure 2:**
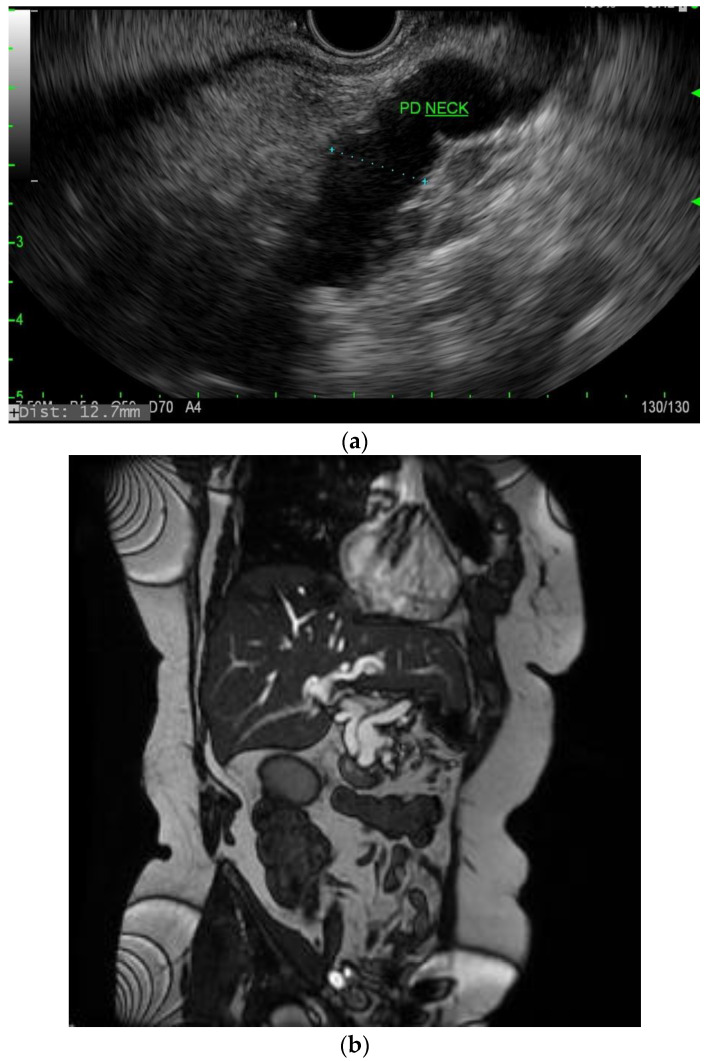
(**a**) EUS image of main duct IPMN; (**b**) MRI image of main duct IPMN.

**Figure 3:**
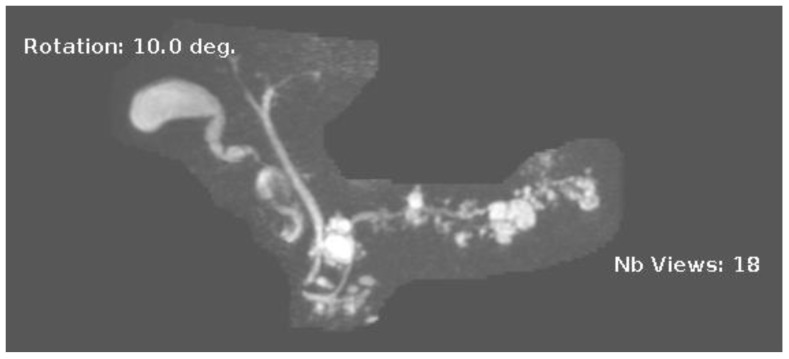
MRI image of side branch IPMN.

**Figure 4:**
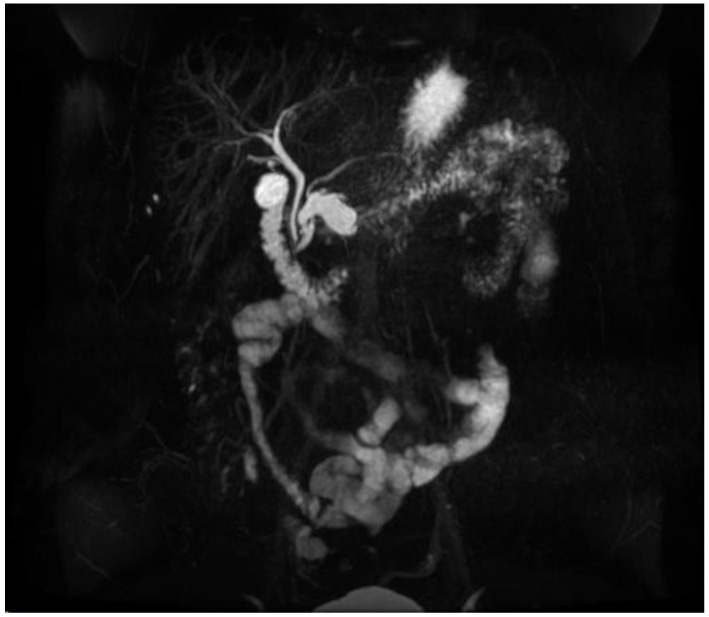
MRI image of mixed IPMN.

IPMNs can be divided into main duct intraductal papillary mucinous neoplasm and branch duct intraductal papillary mucinous neoplasms, with dilation of the main pancreatic duct helping to distinguish between the two. They usually present in the fifth to seventh decade of life and have an equal distribution between males and females [15]. Intraductal neoplasms are typically associated with a dilated main pancreatic duct (Figure 2a,b), whereas the branch duct IPMNs are associated with a dilated pancreatic duct branch or branches (Figure 3). A mixed IPMN is defined as a cystic lesion with ductal communication and main pancreatic duct dilation greater than or equal to 5 mm (Figure 4). Diabetes mellitus (DM) and a family history of pancreatic adenocarcinoma are known risk factors for IPMN, with odds ratios of 1.79 (CI 95%: 1.08–2.98) and 2.94 (CI 95%: 1.17–7.39), respectively [16]. Cytology can show columnar cells with variable atypia and can stain positive for mucin. CEA is usually greater than 200 ng/mL in approximately 75% of lesions, similar to mucinous neoplasms. Main duct IPMNs have a 36–100% risk of malignant potential, compared to a lower risk of 11–30% malignant potential of side branch IPMNs [17].

### 2.3. Serous Cystic Neoplasm

**Figure 5:**
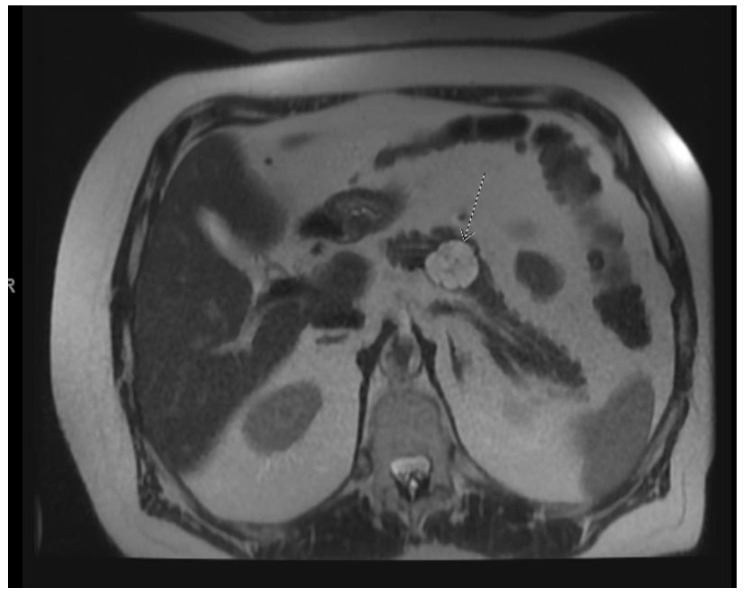
T2 weighted MRI of the abdomen demonstrating a serous cystic neoplasm with a central scar.

SCN usually presents in the fifth to seventh decade of life, and 70% of lesions are found in females [15]. Imaging usually consists of a microcystic or honeycomb-like appearance. A central scar is a characteristic imaging feature but is present in less than 30% of SCAs (Figure 5). There is no malignant potential, and CEA is <5 to 20 ng/mL in the majority of lesions [18]. The aspirate is often thin and bloody [10]. 

### 2.4. Solid Pseudopapillary Neoplasm

SPN usually presents in the second to third decade of life and is found in females 90% of the time [15]. On imaging, it appears as a solid cystic mass and does not typically have a connection with the main pancreatic duct (Figure 1). Typically, the aspirate is bloody, and cytology demonstrates branching papillae with myxoid stroma. Malignant potential is low, cited as 10–15% [10,19]. Surgery is the standard of care for SPN, with excellent reported outcomes and 95% of patients remain disease-free post-surgery [19].

### 2.5. Cystic Neuroendocrine Tumors

**Figure 6:**
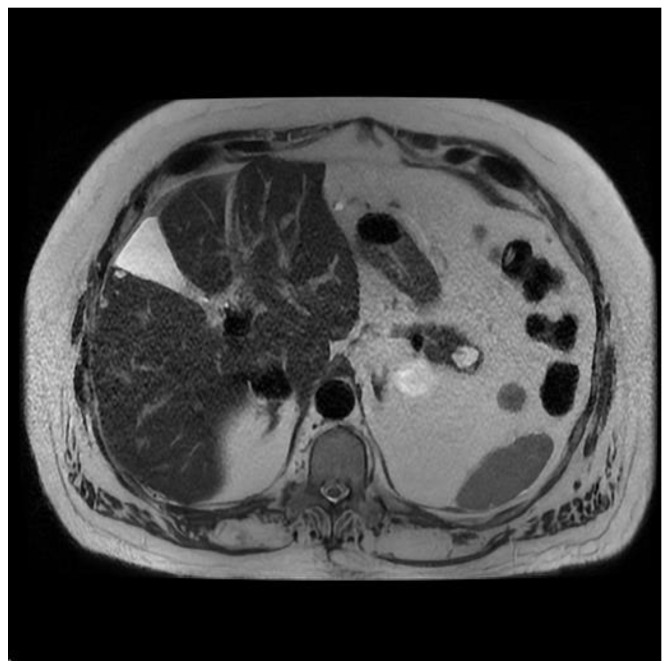
MRI of cNET of the pancreas.

**Figure 7:**
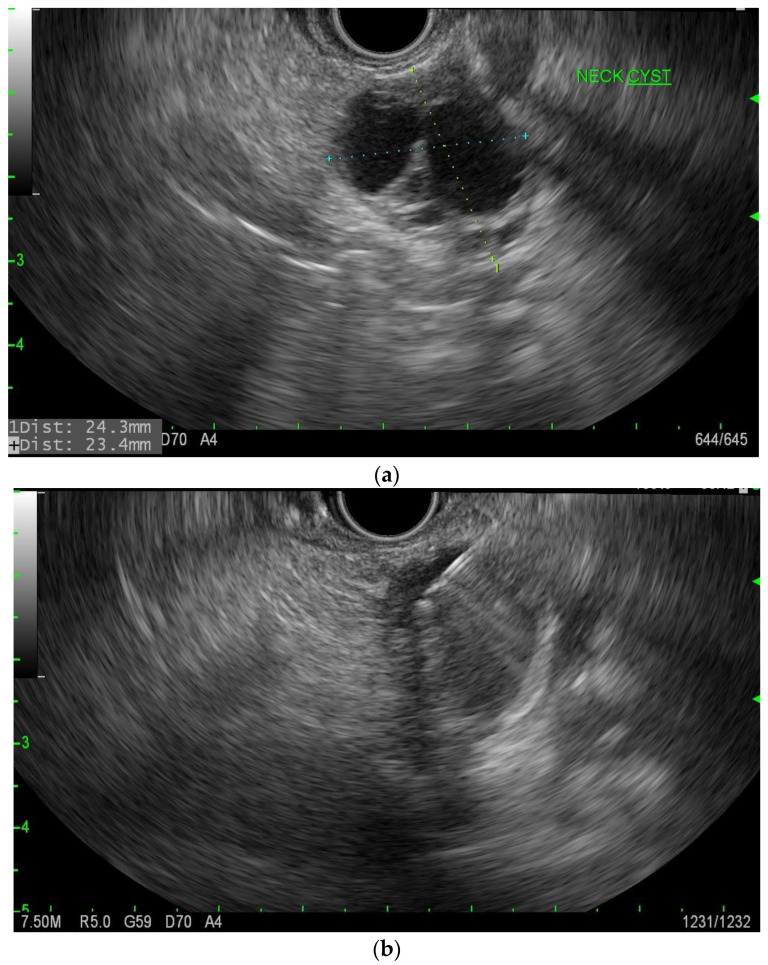
(**a**). EUS of cNET of the pancreas. (**b**). EUS-FNA of cNET of the pancreas.

cNET’s age of presentation varies but usually is in the fifth to sixth decade of life [15]. Imaging usually demonstrates a solid and cystic mass, which is hypervascular (Figure 6). There is usually no connection with the main pancreatic duct, and the malignant potential is 10% [20]. However, pre-operative diagnosis given their small size, and therefore, EUS with FNA has been helpful in that immunostaining of neuroendocrine markers such as synaptophysin and chromogranin-A can differentiate between other types of PCN (Figure 7a,b) [21]. cNET has malignant potential but is very rare in general, and therefore the absolute malignancy risk is difficult to determine. In a systematic review of 64 patients with cystic neuroendocrine tumors, six (9.7%) patients were found to have carcinoma, though these were all resected tumors, so this is likely a higher estimation than the absolute malignancy risk [20]. 

## 3. Diagnosis

The first line imaging for the diagnosis of pancreatic cysts is MRI with MRCP, if not already done. A CT with a dedicated pancreatic protocol is an alternative for patients who are unable to undergo MRI/MRCP; however, MRI with MRCP is more sensitive than CT for identifying a connection with the pancreatic duct, solid components, as well as internal septations, and has the advantage of potentially avoiding contrast [22,23]. EUS morphology has a sensitivity of between 56%–78% and a specificity of 45%–67% for differentiating an IPMN or MCN from other types of cysts [24]. There are specific guidelines for when to employ endoscopic ultrasound (EUS) +/− fine needle aspiration (FNA) for further delineation of the mass if there are clinical or radiological features of concern; however, the data to support EUS as aiding in diagnosis is somewhat limited, with considerable interobserver variability [25,26,27]. With the increasing availability of EUS with the growing number of advanced endoscopists and the relatively low risk of the procedure, it is becoming a useful tool to further characterize cysts and potentially avoid invasive surgery (Supplementary Material Video S1). 

When MRI is compared with EUS, it has been demonstrated to have higher sensitivity for identifying PCNs compared with either imaging technique on its own [28]. The guidelines regarding whether to perform EUS +/− FNA from the American Gastrointestinal Association (AGA), American College of Gastroenterology (ACG), International Association of Pancreatology (IAP), and the European Study Group on Cystic Tumors of the Pancreas all differ, however most recommend EUS if the cyst is greater than 3 cm diameter, have elevated Ca 19-9, pancreatic ductal dilatation, and/or a cyst growth rate greater than 3–5 mm per year (Table 1). 

### 3.1. Cyst Fluid Analysis and Next-Generation Sequencing

If the decision is made to pursue EUS-FNA due to risk factors, both macroscopic and microscopic fluid analysis can aid in the diagnosis of PCN. The string sign is the most sensitive (58%) and specific (95%) indicator of a mucinous PCN [33,34]. The string sign consists of placing a drop of cyst fluid aspirate and stretching it between two fingers before breaking. A mucinous PCN will have a string length greater than 3.5 mm [34]. 

The aspirated fluid is often acellular, and therefore the sensitivity of cytology is low and often non-diagnostic, with a meta-analysis demonstrating a sensitivity of 54% and specificity of 93% for differentiation between mucinous and non-mucinous PCN [35,36]. CEA is more useful for differentiation, as non-mucinous cysts are not able to secrete CEA, positioning the marker as a useful determinator. While there is no pathognomonic correlation of CEA to malignancy, in general, higher CEA indicates a higher likelihood that a cyst is mucinous. Suggested cutoffs have ranged from 192 ng/mL, supported as an internationally accepted cutoff value by European Guidelines, to 20 ng/mL, supported by a systematic review with a sensitivity of 91% and specificity of 93% [35,37]. Intracystic glucose has been suggested to have diagnostic value when CEA levels are indeterminate, as it is associated with lower costs, and a glucose level <25 mg/dL has even been suggested to be more accurate than CEA, with a sensitivity of 88.1% and specificity of 91.2%; however, confirmatory evidence is absent [38].

Testing intra-cystic glucose has several benefits when compared to CEA. Firstly, there is low cost and high availability of glucose testing. More importantly, however, running the test itself requires a small amount of glucose, only 2–50 μL [39]. Often, only small amounts of fluid are able to be aspirated from the cyst via FNA, and therefore, the practical utility of glucose may be greater than CEA, which requires 1000 μL [40]. A hypothesis for why mucinous cysts have lower glucose is that cancer cells require energy in the form of glucose to fuel their rapid division [41]. Multiple studies have shown cyst fluid glucose levels to be lower in mucinous compared with non-mucinous PCNs. A meta-analysis of eight studies including 609 PCNs found that when PCN fluid glucose was compared with PCN fluid CEA, glucose had a higher sensitivity (91% versus 56%) and diagnostic accuracy for detecting mucinous lesions (94% versus 85%), with no difference in specificity between the tests [42]. Low pancreatic cyst fluid glucose (typically <50 mg/dL) was associated with high sensitivity and specificity with significantly improved diagnostic accuracy compared with CEA alone for the diagnosis of mucinous versus nonmucinous pancreatic cystic lesions. Furthermore, in a cohort study of 113 patients, an intracystic glucose level of less than or equal to 41 had a sensitivity of 92% and specificity of 92% for differentiating a mucinous from a non-mucinous cyst, compared to a CEA greater than or equal to 192, which demonstrated a much lower sensitivity of 50%, but a comparable specificity of 92% [43]. 

Lastly, mutated genes found in cystic fluid have been proposed as biomarkers to differentiate between mucinous and non-mucinous PCN, as well as IPMN versus MCN. KRAS and GNAS mutations are highly sensitive and specific for IPMN, but not MCN, and therefore if present, confirm the diagnosis of IPMN [44,45,46]. Mutations or deletions in SMAD4, CDKN2A, TP53, PIK3CA, and/or PTEN are associated with advanced neoplasia; more specifically, the combination of KRAS/GNAS mutations and alterations in TP53/PIK3CA/PTEN have been reported to have an 89% sensitivity and 100% specificity for advanced neoplasia [46]. Further research is necessary to incorporate DNA testing into regular clinical practice; however, these gene analyses should most certainly prompt interdisciplinary discussion with pathologists and surgeons about whether cyst removal is indicated. 

### 3.2. Endoscopic Ultrasound-Guided Through-the-Needle Microforceps Biopsy

Given the variability in sensitivity and specificity of EUS-FNA-guided cyst biopsy and the particularly low sensitivity of cyst fluid cytology, there have been attempts to create devices to obtain more cyst tissue. The Moray microforceps device [US Endoscopy, Ohio, United States] is one of these devices, which can easily pass through a 19-gage needle to obtain a greater amount of tissue when compared to fine needle aspiration. This has the potential to increase diagnostic yield, as well as identify the degree of dysplasia if present. In a systematic review and meta-analysis of 518 patients, there was a significant increase in diagnostic yield of EUS through the needle biopsy (EUS-TTNB) of 79.60% (95% CI, 72.62–85.16; I^2^ = 56.00) when compared to EUS-FNA (OR of 4.79 (95% CI: 1.52–15.06; *p* = 0.007)), as well as increased accuracy (OR 8.69 (95% CI, 1.12 to 67.12; *p* = 0.038)) [47]. The adverse event rate was also low at 1.08%, with the most common adverse events being post-procedure pancreatitis and intracystic bleeding or hematoma, all managed conservatively. Furthermore, in a prospective study of 45 patients with PCN, EUS guided through the needle biopsy was compared to conventional methods of diagnosis (EUS morphology, cross-sectional imaging, and cystic fluid analysis of CEA and amylase/lipase); 10 of 37 cases showed discrepancies between diagnosis by EUS-TTNB and the presumptive diagnosis for identifying the specific types of PCLs, with a diagnostic yield of EUS-TTNB reported at 82% [48]. The adverse event rate was higher at 7% than previously reported studies, potentially due to the greater number of average biopsies taken to improve diagnostic yield. Lastly, in another meta-analysis of 454 patients who underwent EUS-TTNB diagnostic yield was reported as 69.5% (95% CI 59.2–79.7, with a similar adverse event rate of 8.6% (95% CI 4.0–13.1) [49]. In this study, larger cyst size was associated with greater diagnostic yield. This data does suggest the adverse event rate of EUS-TTNB to be higher than EUS-FNA alone, likely associated with its more invasive nature, however with the benefit of potentially higher diagnostic yield. The through the needle biopsy does have the potential to increase the rate of PCN diagnosis, ultimately preventing surgical resection. This may especially be true for larger cysts, where the ability for sampling may be more robust and risk of malignancy is also higher. 

### 3.3. Needle Confocal Laser Endomicroscopy

Confocal laser endomicroscopy (nCLE) enables microscopically detailed real-time visualization of the PCN through a 19-gauge needle used for fine needle aspiration [50]. There are specific findings that are characteristic of certain types of PCN, helping to differentiate between the different lesions. For SCN, a superficial vascular network, or fern pattern, is characteristic [51]. For IPMN, finger-like papillae are usually seen, contrasting MCN, where there is a lack of papillae and rather just layers of the epithelium [52]. The clinical implications of the DETECT study are promising where 30 patients with PCNs underwent nCLE, and the sensitivity of nCLE was 80% (8/10) in18 high-certainty patients, however with post-procedure pancreatitis in two patients (7%) [53]. In a prospective study of 56 patients who underwent nCLE, the findings correlated with the final diagnosis in 77% of cases, compared with 66% for cytology alone [54]. There is more research that needs to be done to confirm the safety and potential benefit to patients of nCLE, and it currently is not recommended by any of the guidelines for routine clinical practice. In the future, EUS-guided through-the-needle biopsies of the PCN wall using microbiopsy forceps may increase the diagnostic yield and further help differentiate non-mucinous from mucinous PCNs and improve presurgical assessment of malignancy risk. 

## 4. Surgical Resection

According to the 2015 AGA [32], 2017 IAP [30], 2018 European [29], and 2018 ACG [10] guidelines, resection is indicated in patients with SPN. According to the 2018 European [29] guideline and the European Neuroendocrine Tumour Society consensus guideline, resection is indicated in patients with cNET >20 mm or if the tumor shows signs of malignant behavior [55]. In patients with MCN or IPMN, the guidelines are not as consistent. Overall, most guidelines determine surgical resection for IPMN to be based on size, pancreatic duct dilatation, symptoms, enhancing mural nodule, and/or cytology being positive for malignancy (Table 2). The IAP Guidelines and European guidelines are generally more aggressive with absolute indications for surgical management, whereas the ACG guidelines recommend referral to an interdisciplinary center for certain criteria, but make no specific recommendations regarding whether to pursue with surgery. 

Surgical resection can be performed with one of three main strategies: pancreatoduodenectomy, also known as the Whipple procedure, distal pancreatectomy, or central pancreatectomy. Decisions about the type of surgery depend on where the nodule is located. The complications and morbidity associated with these surgeries are high, with complication rates for Whipple estimated to be about 40%, distal pancreatectomy at 25%, and central pancreatectomy at 51% [56,57]. Therefore, the guidelines are aimed at reducing morbidity while preventing potential terminal cancer. Absolute indications for surgery are indicated for each guideline are outlined in Table 2, largely based on cyst size, symptoms, pancreatic duct enlargement and/or the presence of a mural nodule. Relative indications for cyst removal indicated in each guideline are outlined in Table 3. 

### 4.1. Cyst Size

Cyst size greater than 3 cm has been associated with a greater chance of malignancy. When compared to other variables, such as the presence of mural nodules and/or pancreatic duct dilation on multivariate analysis, cyst diameter has been shown to be the only independent predictor of malignancy [58]. However, the data is somewhat conflicting on exactly how predictive size is regarding malignancy risk. The odds ratio of IPMN cyst size greater than 3 cm for underlying malignancy has been reported to be as low as 2.97 (1.82–4.85) to as high as 62.4 (30.8–126.3) [59,60]. In a retrospective study of 63 patients with MCN, with cyst sizes ranging from 0.5 to 18.5 cm with a median size of 3.5 cm, tumor size did not correlate to a final diagnosis of malignancy [61]. Contrastingly, in a retrospective study including eight academic centers consisting of 349 patients with MCN, increased radiographic size of the MCN (OR, 1.17; 95% CI, 1.08–1.27; *p* < 0.001) was independently associated with malignancy risk [62]. The discrepancy in the guidelines is reflective of the conflicting available data. The 2018 European guidelines are generally less aggressive than the 2015 AGA guidelines and 2018 ACG guidelines. 2018 European guidelines recommend surgery for MCN with a cyst diameter greater than or equal to 4 cm, as well as if there is any enhancing mural nodule, whereas 2017 IAP and AGA guidelines recommend surgery for any MCN regardless of size. Lastly, AGA and ACG offer no recommendation for surgery based on MCN size, and ACG recommends just referral to EUS-FNA multidisciplinary team if IPMNs or MCNs ≥ 3 cm.

### 4.2. Pancreatic Ductal Dilatation

Similar to cyst size, there is also a discrepancy between studies assessing the relationship between pancreatic ductal (PD) dilation and malignancy risk. A meta-analysis including 6301 patients with IPMN found a sharp change in the size of the main pancreatic duct (MPD) was strongly predictive of high-grade dysplasia or pancreatic cancer (OR 7.41, CI 2.49–22.06) [63]. Additionally, a meta-analysis with 328 patients found a PD greater than or equal to 6 mm had an odds ratio (OR) of 7.27 (95% CI, 3.0–17.4) for developing malignancy [59]. Another meta-analysis with 609 patients who underwent surgery for their PCN found a much lower odds ratio of 2.38 (95% CI, 0.71–8.00) [60]. While there is definitely evidence to suggest a dilated PD >5 mm is associated with malignancy and >10 mm with an even higher association, the degree to which this association exists is unknown [64]. 

Interestingly, the 2015 AGA guidelines are more aggressive about IPMN surgical resection, recommending this in PD greater than or equal to 5 mm and a solid component, whereas the 2017 IAP and 2018 European Guidelines recommend surgery if PD is greater than or equal to 10 mm. ACG recommends referral to a multidisciplinary team if MPD dilation >5 mm, focal or dilation of PD for MD IPMN. 

### 4.3. Symptoms

For MCN, the only guideline for surgical resection that includes symptoms is the 2018 European Guidelines, with jaundice, acute pancreatitis, and new-onset diabetes mellitus. As for IPMN, the 2017 IAP guideline and the 2018 European Guidelines recommend surgery for tumor-related jaundice. For ACG, referral to a multidisciplinary team is recommended for evaluation of surgery with jaundice or acute pancreatitis secondary to the cyst.

### 4.4. Enhancing Mural Nodule

A meta-analysis including 2297 resected IPMNs with a mural nodule found the presence of an enhancing mural nodule had a positive predictive value of 62% for the presence of advanced neoplasia at final pathology, suggesting that mural nodules are indicative of a malignant component of the cyst (Supplementary Material Video S2) [65]. 2017 IAP guidelines recommend surgery for enhancing mural nodule presence in IPMN, as well as 2018 European guidelines, 

ACG recommends multidisciplinary team referral for the presence of a mural nodule or solid component within the cyst.

**Table 3:** Relative Indications for Surgical Resection of Pancreatic Cysts.

2015 AGA [32]	MCN	-
	IPMN	-
2017 IAP [30]	MCN	-
	IPMN	Cyst growth rate >5 mm/year. Increased levels of serum CA19-9 PD dilatation between 5 and 9 mm Cyst diameter ≥30 mm Acute pancreatitis (caused by IPMN) Enhancing mural nodule (<5 mm) Abrupt change in diameter of PD with distal pancreatic atrophy Lymphadenopathy Thickened or enhancing cyst walls
2018 European [29]	MCN	-
	IPMN	Growth rate ≥5 mm per year Increased levels of serum CA19-9 (>37 U/mL) PD dilatation between 5 and 9.9 mm Cyst diameter ≥40 mm New-onset diabetes mellitus Acute pancreatitis (caused by IPMN) Enhancing mural nodule (<5 mm)
2018 ACG [10]	MCN	-
	IPMN	Jaundice or acute pancreatitis secondary to the cyst Significantly elevated serum CA 19-9 level Presence of a mural nodule or solid component within the cyst PD dilation >5 mm Focal dilation of PD for MD-IPMN or an obstructing lesion ≥3 cm Presence of HGD-IPMN or pancreatic cancer on cytology

## 5. Surveillance 

There is evidence for those who do undergo surgery for high-grade dysplasia or very early pancreatic cancer have improved survival rates, suggesting that early detection and intervention may be beneficial; however, mortality benefit and/or pancreatic cancer prevention have not been demonstrated with surveillance of pancreatic cysts [66]. There has not been a robust time course of data collection in order to perform a comprehensive analysis. Therefore, ensuring patient and provider conversation with informed consent and discussion of surgical candidacy is essential before enrolling a patient into an imaging surveillance program. Surveillance should only be offered to patients who are physically fit for surgery and continue to be so throughout the surveillance interval. 

### 5.1. Active Surveillance of Non-Resected PCNs 

No surveillance is needed in asymptomatic patients with SCN, as the risk of malignancy is extremely low [18]. Surgery is only recommended in patients with symptomatic SCN related to compression. According to the European guideline, surveillance is recommended for patients with asymptomatic cystic pancreatic neuroendocrine tumors <20 mm in size, however, the method and interval of surveillance are not specified [29]. A meta-analysis comparing 152 cystic versus 915 solid cNETs concluded that cystic cNETs tend to be biologically less aggressive and cNETs have an approximately 20% risk of malignancy, with a 5-year overall survival of 87–100%, reassuringly supporting a less aggressive management strategy [20].

The 2017 IAP and 2018 ACG differentiate surveillance intervals of IPMN and MCN based on the size of the cyst, whereas the 2015 AGA and 2018 European Guidelines do not. The guidelines are detailed below in Table 4. There is no evidence to support when to stop the surveillance of PCN. AGA has the least conservative recommendation in terms of stopping surveillance, and they plan to stop after 5 years if the cyst is stable, whereas most of the other guidelines recommend lengthening intervals. The other guidelines recommend considering comorbidities and, if at any point a patient would not be fit for surgery, stopping surveillance after a period of stability.

### 5.2. Surveillance after Surgical Resection of PCNs

The management of resected PCNs largely differs depending on whether invasive cancer was seen on the final pathology of the resected specimen. Evidence has supported no further surveillance after the resection of MCN, even with low- or high-grade dysplasia, but without invasive cancer, given a systematic review of 773 patients demonstrated no risk of recurrence [67]. In contrast, IPMN requires lifelong surveillance after partial pancreatectomy, given the higher risk of recurrence [68]. The recommended interval differs depending on the grade of dysplasia found and family history. In a study of 130 patients followed for a median of 38 months after resection of IPMN, the estimated chances of developing invasive pancreatic cancer were 0%, 7%, and 38% at 1, 5, and 10 years, respectively. A family history of pancreatic cancer was predictive of developing a new IPMN (23% vs. 7% (*p* < 0.05)) [69]. Similarly, in a study of 126 patients who underwent IPMN resection and were followed for a median of 9.5 years, a family history of pancreatic cancer (hazard ratio 3.05) and high-grade IPMN (hazard ratio 1.88) were risk factors for recurrence [70]. If high-grade dysplasia is found or main duct involvement, 2018 European guidelines recommend imaging every 6 months for the first 2 years, followed by yearly surveillance, whereas 2017 IAP guidelines recommend imagining at least twice a year for patients with high-grade dysplasia, non-intestinal subtype, and/or family history of pancreatic cancer. In low-grade or borderline-grade dysplasia, 2018 European guidelines require imaging every 6 months for 1 year, then yearly, and similarly, the 2017 IAP guidelines recommend follow-ups every 6–12 months [29,30].

## 6. Future Directions

Increasingly, research is being conducted to evaluate potential treatments for PCN other than surgery, with the goal of less morbidity and mortality. There have now been numerous trials evaluating the safety of both EUS-guided intracystic alcohol ablation and taxol ablation. In a systematic review of six studies (N = 207 patients) evaluating alcohol lavage and eight studies (N = 347 patients) evaluating paclitaxel-based regimens, the rate of complete cyst resolution was much higher in paclitaxel-based regimens, 63.6% compared to alcohol lavage, 32.8%. Adverse events were also lower in paclitaxel-based regiments, 15%, compared to alcohol lavage 21.7%, with most consisting of abdominal pain, pancreatitis and intracystic bleeding, in that order [71]. Additionally, a recent early-phase trial with nineteen subjects receiving two dosing regimens of large surface area microparticle paclitaxel (LSAM-PTX) achieved a resultant cyst volume reduction in 70.6% of patients without any significant toxicities [72]. Given the higher efficacy and lower side effects of EUS-guided taxol cyst ablation, this may be a revolutionary future treatment; however, more studies need to be done to study both its efficacy and safety. 

As evidenced by the discrepancies between the major guidelines for PCN surveillance intervals and indications for EUS and/or surgery, quality evidence to support PCN management is lacking. The data to support the risk of progression of PCN to malignancy is conflicting and differs significantly between each PCN subtype. However, given the slow progression and high mortality rate of pancreatic cancer, clinicians have an opportunity to potentially identify and treat this disease early, with the goal of ultimately preventing morbidity and mortality. Given the complexity of managing these patients, referral to a high-volume multidisciplinary center is extremely important, and with the rise of telemedicine during the COVID-19 pandemic, the barrier of transportation may be eased [73]. Given that most guidelines recommend lifelong surveillance, barriers to accessing tertiary academic centers, specifically the volume and wait time of referrals, must be addressed. Lastly, large-scale prospective studies are needed to aid the future determination of whether these screening strategies prevent morbidity and mortality from pancreatic cancer. 

## Figures and Tables

**Table 1:** Indications for Endoscopic Ultrasound of Pancreatic Cystic Neoplasm.

**Society**	American College of Gastroenterology (2018) [10]	European Guideline (2018) [29]	International Association of Pancreatology (2017) [30]	American Society of Gastrointestinal Endoscopy (2016) [31]	American Gastrointestinal Association (2015) [32]
**Cyst Diameter**	>3 cm diameter	N/A	>3 cm diameter	>3 cm diameter	>3 cm diameter
**Pancreatic Duct Dilatation**	Yes	N/A	Yes, also an abrupt change in the caliber of the pancreatic duct with distal pancreatic atrophy	Yes	Yes
**Nodule**	Yes	N/A	Enhancing mural nodule <5 mm	Presence of epithelial nodule or suspicious mass lesion	Yes
**Rate of Growth**	Cyst growth rate greater than 3 mm per year	N/A	Cyst growth rate greater than or equal to 5 mm over 2 years	N/A	
**Biomarker**	Elevated Ca 19-9	N/A	Elevated Ca 19-9		N/A
**Symptoms**	Jaundice or Acute Pancreatitis	N/A	N/A	N/A	N/A
**Additional Recommendations**	If not clearly an IPMN or MCN based on cross-sectional imaging, EUS-FNA is recommended.	EUS-(FNA) should only be performed when the results are expected to change clinical management or PCN has either clinical or radiological features of concern identified during the initial investigation or surveillance.	Thickened/enhancing cyst walls, Lymphadenopathy	EUS-FNA is optional for cysts <3 cm without other indications for performing EUS-FNA.	

**Table 2:** Absolute Indications for Surgical Resection of Pancreatic Cysts.

Guideline	Cyst Type	Size	Pancreatic Duct Dilatation	Symptoms	Enhancing Mural Nodule	Cytology Positive for Malignancy
2015 AGA [32]	MCN	All	All	All	All	All
	IPMN	-	>5 mm and solid component	-	-	Yes
2017 IAP [30]	MCN	All	All	All	All	All
	IPMN	≥5 mm	≥10 mm	Jaundice	Yes	Yes
2018 European [29]	MCN	≥40 mm	-	Jaundice, acute pancreatitis, new-onset diabetes mellitus	Yes	-
	IPMN	-	≥10 mm	Jaundice	Yes ≥5 mm	Yes
	cNET	>20 mm	-	-	-	Yes
2018 ACG [10]	MCN	-	-	-	-	-
	IPMN	-	-	-	-	-

**Table 4:** Imaging Surveillance Interval of Non-Resected Pancreatic Cysts.

Guideline	Cyst Type	Cyst Size	Surveillance Interval	Imaging Modality	When to Lengthen Interval or Stop If Stable
2015 AGA [32]	IPMN	<30 mm	Yearly for 1 year, then every 2 years	MRI with MRCP	Stop after 5 years
2017 IAP [30]	IPMN	<10 mm	6 months and then every 2 years	CT or MRI with MRCP	Lengthen interval after 3 years
		10–20 mm	Every 6 months for one year and then yearly for every 2 years	CT or MRI with MRCP	Lengthen interval after 3 years
		20–30 mm	3–6 months, then yearly	EUS alternating MRI with EUS	Lifelong surveillance
2018 European [29]	MCN and IPMN	<40 mm	Every 6 months for 1 year, then yearly	Ca-19-9, EUS and/or MRI	Lifelong surveillance
	cNET	<20 mm	No guidance on interval or screening modality		
2018 ACG [10]	MCN and IPMN	<10 mm	Every 2 years	MRI	Lifelong surveillance
		10–20 mm	Yearly for 3 years, then every 2 years	MRI	Lifelong surveillance
		20–30 mm	Every 6–12 months for 3 years, then yearly	MRI or EUS then MRI	Lifelong surveillance

## Data Availability

No new data were created or analyzed in this study. Data sharing is not applicable to this article.

## Supplementary Materials

The following supporting information can be downloaded at: https://www.mdpi.com/article/10.3390/diagnostics13020207/s1, Video S1: EUS of a serous cyst, Video S2: EUS of a cyst with a mural nodule.
